# Fake news on Social Media: the Impact on Society

**DOI:** 10.1007/s10796-022-10242-z

**Published:** 2022-01-19

**Authors:** Femi Olan, Uchitha Jayawickrama, Emmanuel Ogiemwonyi Arakpogun, Jana Suklan, Shaofeng Liu

**Affiliations:** 1 Newcastle Business School, Northumbria University, Newcastle Upon Tyne, UK; 2 School of Business and Economics, Loughborough University, Loughborough, UK; 3 NIHR Newcastle IVD Co-operative Translational and Clinical Research Institute, Newcastle University, Newcastle Upon Tyne, UK; 4 Plymouth Business School, University of Plymouth, Plymouth, UK

**Keywords:** Fake news, Misinformation, Societal acceptance, Social media, Societal values, True news

## Abstract

Fake news (FN) on social media (SM) rose to prominence in 2016 during the United States of America presidential election, leading people to question science, true news (TN), and societal norms. FN is increasingly affecting societal values, changing opinions on critical issues and topics as well as redefining facts, truths, and beliefs. To understand the degree to which FN has changed society and the meaning of FN, this study proposes a novel conceptual framework derived from the literature on FN, SM, and societal acceptance theory. The conceptual framework is developed into a meta-framework that analyzes survey data from 356 respondents. This study explored fuzzy set-theoretic comparative analysis; the outcomes of this research suggest that societies are split on differentiating TN from FN. The results also show splits in societal values. Overall, this study provides a new perspective on how FN on SM is disintegrating societies and replacing TN with FN.

## Introduction

In cascading news and sensitive information, the fundamental principles are embedded in the concepts of truth as well as the theories of accuracy in communication (Brennen, 2017; Dwivedi et al., 2018; Orso et al., 2020; Pennycook et al., 2020). However, in the past five years or so, social media (SM) has redefined the structure, dimensions, and complexity of the news (Berkowitz & Schwartz, 2016; Copeland, 2007; Kim & Lyon, 2014). The impact of SM, specifically on political affairs, has been attracting more interest, as SM platforms, notably Twitter, Facebook, and Instagram, enable the broad sharing of information and news (Vosoughi et al., 2018). In addition to providing information, another main purpose of SM is to enable people to engage in social interaction, communication, and entertainment (Hwang et al., 2011; Kuem et al., 2017). In particular, many SM posts are looking for support, where reposting aims to spread messages via the multiplicative effect. Consequently, this study purpose is to address the research problem and gap which suggest that SM platform providers are doing little in tackling the spread and cascading of FN on SM.

By providing unlimited access to a large amount of information, people can share different beliefs and values (George et al., 2018; Kim et al., 2019; Rubin, 2019). However, the risks and implications of this new resource remain unclear to most of the population. One such risk is fake news (FN). FN, although unvetted, has a credible and professional appearance, ensuring that people cannot always distinguish it from true news (TN) (Kumar et al., 2018). The effects of FN cut across the society, for example, the spread of FN on SM determines how governments, organizations, and people respond to events in the society. Majority of FN is targeted to a specific sample of the population with the aim of promoting a certain ideology by stimulating strong beliefs and polarizing society (Chen & Sharma, 2015). According to Kumar et al. (2018); Lundmark et al. (2017); Tandoc et al. (2019), a periodic review of FN on SM is thus required to limit discord and violence by groups or individuals in society.

FN has become a major part of SM, raising doubts about information credibility, quality, and verification. Studies investigating the influence of FN on SM have appeared in various fields such as digital media, journalism, and politics; however, in-depth analyses of the impact of FN on society remain scarce. Furthermore, despite the growing body of research on FN and SM —a significant factor in the fight against FN —(Tandoc et al., 2018), an adequate review of the impact of FN in SM on society is also lacking.

Hence, The aim of this study is to explore the role of SM platform providers in reducing the spread of FN in the society, as the research gap identified from previous studies (Kim & Dennis, 2019; Kim et al., 2019; Knight & Tsoukas, 2019; Roozenbeek & van der Linden, 2019) on the limited research on the impact of FN on the society, leading to this study finding answers to the following research questions (RQs):

- RQ1. Why is FN cascading impacting negatively on the society?
- RQ2. Are the big SM organizations taking actions in reducing FN cascading?

Based on the foregoing, this study provides a holistic view of the three focus areas (FN, SM, and societal acceptance) by reviewing research publications, case studies, and experts’ opinions to produce a conceptual framework, an insightful and comprehensive meta-framework. This study then analyzes the associations among the three distinct fields from theoretical and practical perspectives. These associations derived from the literature are tested using an analytic technique called fuzzy set analysis to show if they are supported, thereby indicating society’s efforts to combat FN. We find that people’s interpretations of what is TN or FN affect societal efforts to reduce the spread of FN.

The findings of this study contribute to research on FN on SM, specifically looking at societal impacts. They provide experts and researchers in these fields with insights into how communities are effectively combating the spread of FN and how to implement the useful ideas from this research to strengthen the inputs in tackling FN on SM. Further, the findings of this research not only provide support for the associations but demonstrate a model for societal strategies to manage the spread of FN as well as fact-checking and information verification, thus equipping society with the tools to recognize the differences between FN and TN.

The remaining sections in this study are organized as follows: the theoretical development of the conceptual meta-framework explains the literature for the concept of FN, SM, and societal acceptance. This is followed by researched method section that describes the data, analysis and presents the results of the study. Further, there is a discussion section on the results, implications of this study for research, practice, and the society, finally limitations and future research.

## Theoretical Development of the Conceptual Meta-Framework

FN is shaped to replicate TN by mimicking its characteristics (i.e. accuracy, verifiability, brevity, balance, and truthfulness) to mislead the public (Han et al., 2017; Kim & Dennis, 2019; Kim et al., 2019). FN is not a new phenomenon, according to Burkhardt (2017), FN can be traced back to at least Roman times when the first Roman Emperor had to announce fake news to encourage Octavian to destroy the republican system. During the Roman period, there was no way of verifying and validating the authenticity of news, as challenging authority was classed as treason. The 20th century heralded a new era of numerous one-to-many communication modes such as newspapers, radio stations, and television stations, marking the beginning of misinformation in news (Aggarwal et al., 2012; Kim & Dennis, 2019; Kim et al., 2019; Knight & Tsoukas, 2019; Manski, 1993; Preti & Miotto, 2011; Roozenbeek & van der Linden, 2019). With the emergence of multimedia corporations, the content of FN has been gaining new audiences (Oestreicher-Singer & Zalmanson, 2013), and the arrival of the Internet towards the end of the century improved the phenomenon of FN (Kapoor et al., 2018). As technology advanced in the 21st century, SM arrived, multiplying the dissemination of FN using both one-to-many and many-to-many strategies.

### Understanding FN

FN content, which is divided into individual opinions and scientific consensus on trending issues such as COVID-19, evolution, and climate change, has long existed (Knight & Tsoukas, 2019). However, constant changes in political strategies have fundamentally impacted how information is defined, viewed, and interpreted at all levels of communication (Massari, 2010). Aggarwal and colleagues argued that incorrect scientific, political, and belief-oriented information has significant causes and consequences on individuals that are more politically inclined and those aiming to drive their ideas to wider society (Aggarwal et al., 2012). Therefore, individuals actively seeking information are united in their pursuit of knowledge and political action (Aggarwal & Singh, 2013). It is impossible to change their values and beliefs, abandon old ways and accept the fact-checked news, new methods to enlightening individuals or people with similar beliefs to adopt new states to a degree of news verification and validation (Cao et al., 2015; Centeno et al., 2015; Kim & Lyon, 2014).

As FN is fundamentally built on untraced and misleading phenomena, experts and researchers have noted a rising interest in the development of fact-checking tools to spot the spread of FN content in society (Berkowitz & Schwartz, 2016; Hwang et al., 2011; Miranda et al., 2015; Miranda et al., 2016). However, despite the large investment in innovative tools for identifying, distinguishing, and reducing factual discrepancies (e.g., ‘Content Authentication’ by Adobe for spotting alterations to original content), the challenges concerning the spread of FN remain unresolved, as society continues to engage with, debate, and promote such content (Kwon et al., 2017; Pierri et al., 2020). Indeed, the gap between fact-checking and the fundamental values and beliefs of the public discourages people from promoting fact-checking rather than accepting the dangers of FN (Kim & Lyon, 2014; Lukyanenko et al., 2014). Therefore, these tools do little to reduce the spread of FN in practice.

### SM and Society

SM provides an environment in which individuals can exchange personal, group, or popular interests to build relationships with people that have similar and/or diverging beliefs and values. For example, most people of a particular age group share similar interests courtesy of growing up in the same era (Gomez-Miranda et al., 2015; Lyon & Montgomery, 2015; Miller & Tucker, 2013; Nerur et al., 2008). People’s characteristics are often inherited from educational institutions, communities, and family lifestyles (Matook et al., 2015). Further, certain age groups continue to hold onto specific values and beliefs, as reflected in the public’s response to the 2016 and 2020 U.S. presidential election and the 2019 UK general election (Prosser et al., 2020; Wang et al., 2016). Accordingly, Venkatraman et al. (2018) argued that values and beliefs are passed down through family generations, making it possible for a group in society to continue to hold onto specific philosophies.

SM plays an important role in helping people reconnect with friends and families as well as find jobs and purchase products and services (Kim & Dennis, 2019; Leong et al., 2015; Lyon & Montgomery, 2015; Miller & Tucker, 2013; Nerur et al., 2008; Pierri et al., 2020). SM platforms are also channels for recruiting interested parties for the continuity and propagation of a long-held ideology. Moreover, people with common demographic attributes use the instant messaging services on SM to communicate more than those without such shared demographics (Baur, 2017). SM platforms are thus online services that mirror real-world activities (e.g., dating services from Facebook, live Instagram feeds from parties).

The societal acceptance strategy can reduce the spread of FN (Haigh et al., 2018; Lundmark et al., 2017; Lyon & Montgomery, 2015; Miller & Tucker, 2013; Nerur et al., 2008; Sommariva et al., 2018). However, the expansion of multiple access points for information and news sharing on SM platforms contributes more to the spread of falsity than reducing its impact. Nevertheless, societal acceptance is considered to be a game-changer for controlling the spread of FN by SM (Egelhofer & Lecheler, 2019). Some empirical studies have analyzed the spread and flow of FN online (Garg et al., 2011; Gray et al., 2011), but little research examines how human judgment can differentiate truth from falsity. To reduce the spread of FN in society, it is important to understand the triangle of FN, the relationships between the constructs from each circle, and the associations that bind the circles, and then analyze the strength of the relationship (Chang et al., 2015; Chen & Sharma, 2015; Matook et al., 2015).

### Meta-framework on the Impact of FN

This study developed a meta-framework based on the literature on FN, SM, and societal acceptance. Each of these perspectives, depicted as circles in the meta-framework, discusses the constructs that contribute to defining the clusters in theory. The constructs that then emerge from each perspective are the foundation for the meta-framework discussing the relationships among their associations. This study further develops notations to define the associations. By combining the three defined circles, these perspectives provide a new theoretical framework, as previous studies have shown that feasibilities to conceptualize phenomenon are at a wide spectrum (Table 1).

**Table 1:** Summary of the key theoretical studies

Studies	Context of FN, SM, and SA	Research aims	Summary/main outcome	Relationship to FN, SM, and SA	Benefit to FN, SM, and SA	
(Burkhardt, 2017; Kapoor et al., 2018; Kim et al., 2019; Pan et al., 2017; Venkatraman et al., 2018; Vosoughi et al., 2018)	Verification/fact checking	Establishing a system or processes dedicated to authenticating the content in the news and its intentions	Comparing multiple platforms, users; and FN; evaluating and analyzing data using specific analytic techniques to derive results	Finding associations from the FN literature to support the meta-framework in this research	Supporting the investigation of the relationships defined regarding the attributes in the FN construct	
(Brummette et al., 2018; Chang et al., 2014; George et al., 2018; Kim & Dennis, 2019; Kwon et al., 2017; Leong et al., 2015; Sommariva et al., 2018)	SM platforms	Understanding the operations of platforms, analyzing the spread and cascading of news, and observing patterns in users’ consumption behavior	Applying key fact-checking and cascading indicators to evaluate FN and content on SM platforms	Finding associations from the SM literature to support the meta-framework in this research	Supporting the investigation of the relationships defined regarding the attributes in the SM construct	
(Barrett et al., 2016; Brennen, 2017; Burkhardt, 2017; Fang et al., 2013; Kapoor et al., 2018; Lazer et al., 2018; Posetti & Matthews, 2018; Tandoc et al., 2018)	Society	SA strategies, models, and implementations incorporating news content, content processes, and transmission	This holistic approach compares traditional news processes with modern news processes as well as traditional news verification and validity with modern verification and validity	Finding associations from the SA literature to support the meta-framework in this research	Supporting the investigation of the relationships defined regarding the attributes in the SA construct	
(Ragin, 2013; Ragin & Pennings, 2005)	Fuzzy set	A set theoretic technique designed for set theory analysis by creating patterns of attributes defined by numerous features and generating outcomes on the construction of relationships	Complementarity and equifinality testing by generating consistency and solution coverage	The combination system supports relationships among the FN, SM, and SA constructs	A holistic approach targeting new attributes in the three constructs’ mapping to establish relationships among collecting data, testing theory, and producing outcomes	
(Chen et al., 2015; Kumar et al., 2018; Kwon et al., 2017; Roozenbeek & van der Linden, 2019; Venkatraman et al., 2018)	Technology	Development of a hybrid intelligent system that supports fact-checking and uses SM and information management	The system was empirically assessed with SM platforms’ decision-makers. The results showed that the hybrid system supported strategy development	An understanding of how technology is supporting the fight against the spread of FN and challenges in its use	Society helping reduce the spread and cascading of FN; understanding fact-checking and verifying news	

Note: SA = Societal acceptance

**Fig. 1:**
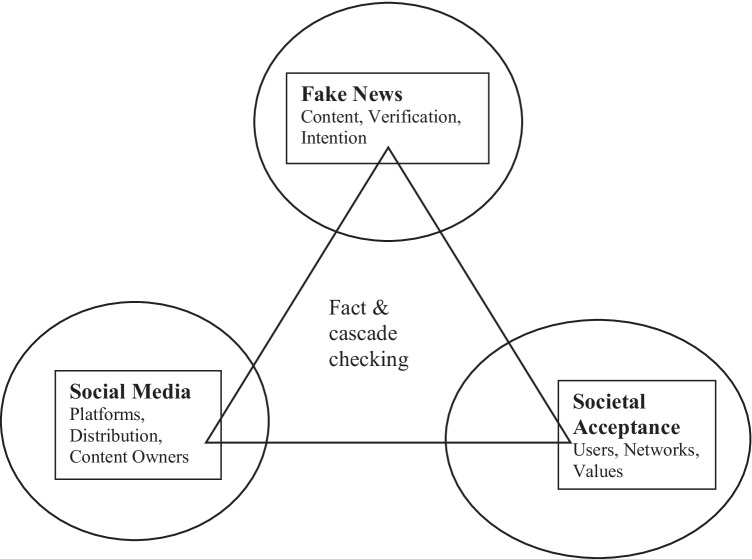
Fake news triangle

This study adopted the epidemiological model as a suitable theory for discussing the meta-framework perspectives. In particular, it employed the conceptual model of the disease triangle. In the 1960 s, the disease triangle was developed by George McNew to understand the pathology and epidemiology of plants and their diseases (Scholthof, 2007). This model stated that for a disease to manifest, three fundamental elements are required: the environment; the infectious pathogen that carries the virus, bacteria, or other micro-organisms; and the host. In this study, FN is defined as an ‘infectious pathogen’, as it is an epidemic that consists of varieties of fake news (Pan et al., 2017). According to Scholthof (2007), the environment determines whether the infection can be controlled; here, as shown in Fig. 1, SM is conceptualized as the environment, the hosts are the readers, individuals, and society.

SM as an environment for cascading of FN has a structure (Chen et al., 2015; Miller & Tucker, 2013; Scholthof, 2007). The aim of the SM structure is to generate contents that attract millions of views by re-sharing news or information targeting a set of specific viewers. As the contents are shared and attained a viral status in the society, SM organizations are leveraging increased profits (Mettler & Winter, 2016). Primarily, SM structure is designed on contents ranking system constructed by algorithm ranking techniques, the method of data management and significance leveling in data priority (Hamamreh & Awad, 2017). News and information are ranked in a methodological order that links constructing a natural distribution by connecting between nodes of the SM (Gerlach et al., 2015; Matook et al., 2015). To understand the ranking system in SM, each node is assigned a unique code by creating iterative process of weights in network, these weights are assigned according to the content structure of the SM node (Brennen, 2017; Burkhardt, 2017; Chen, 2018). According to Brennen (2017); Burkhardt (2017); Chang et al. (2014); Chen (2018); Maier et al. (2015); Massari (2010), SM as the environment for infectious contents like FN comprises of communication channels such as websites, mobile applications, and platforms that facititate relationship forming among users of contents with similar interest. Hence, the relevance of SM to various aspects of life is of high singficance to users, government policies, and the economy.

This is somewhat consistent with the argument of the Director-General of the World Health Organization (WHO) – Tedros Ghebreyesus – at a foreign policy and security expert submit held in Germany in February 2020 (Union, 2020, May 19). Tedros argued that as the world continues to grapple with Covid-19 contagion, an ‘infodemic’ is emerging as FN continues to “spread faster and more” than Covid-19 (Africe, 2020). Given the speed of the spread of FN, infodemic can hinder the effectiveness of public health response while propagating confusion and distrust in the society.

As shown in Fig. 1, the hosts interact with those who have similar interests in their SM groups or forums and thus recruit new believers to the environment (Haigh et al., 2018; Humprecht, 2019a; Mettler & Winter, 2016; Roozenbeek & van der Linden, 2019; Rubin, 2019). These communities continue to grow as positive social networks expand. With the power of SM platforms, new groups are created that have a similar agenda, improving social learning and opportunities using SM platforms’ tools (Kwon et al., 2017). One of the purposes of these strategies and networks is to clamp down as quickly as possible on people perceived as outsiders that may uncover or expose their content and philosophies.

## Research Method

### Research Design and Data Collection

This study carried out a longitudinal survey with online participants to test the relationships and associations in the proposed meta-framework. A cross-sectional online survey was conducted in 2019, survey was conducted using stratified sampling, with participants divided into groups based on their demographics, proficiency of using SM platforms, and interest in news and current affairs online. Table 2 shows participants’ profiles in terms of their gender, age, location, SM usage, and SM experience. The questionnaire was designed through the research gap and literature.

**Table 2:** Participants’ profiles

	No.	Percentage		No.	Percentage
Sex			SM platform usage		
Male	137	38.5	Once a week	4	1.0
Female	219	61.5	2–4 times a week	7	2.1
			5–6 times a week	19	5.2
Age			Once a day	56	15.8
18–24	75	21.2	2–3 times a day	81	22.9
25–34	111	31.1	4–5 times a day	88	24.6
35–44	85	23.7	More than 5 times a day	101	28.4
45–54	70	19.6			
55–64	10	2.9	SM platform experience		
65 or above	5	1.5	Less than a year	27	7.6
			1–2 year(s)	37	10.5
Location			3–4 years	65	18.2
Africa	45	12.5	5–6 years	81	22.7
Antarctica	21	5.9	7–8 years	79	22.3
Asia	41	11.6	9–10 years	38	10.6
Australia plus Oceania	45	12.7	More than 10 years	29	8.1
Europe	92	25.8			
North America	105	29.4			
South America	7	2.1			

This study distributed the questionnaire to 2234 active engaging participants and received 546 surveys which included both partial and completed questionnaire, which accounts for a response rate of 24%, demonstrating that the response rate is consistent with previous studies (Arshad et al., 2014; Klashanov, 2018; Malik et al., 2020). This study sample size consists of participants from across the global, with North America accounting for 29% of the total survey which make up for the largest share in terms of participant size. Experience of using SM platforms show that 28% of the participants engage more than 5 times daily on the platforms while 22.7% accounting for participants with 5 to 6 years working the SM platforms.

### Analytical Technique

**Fig. 2:**
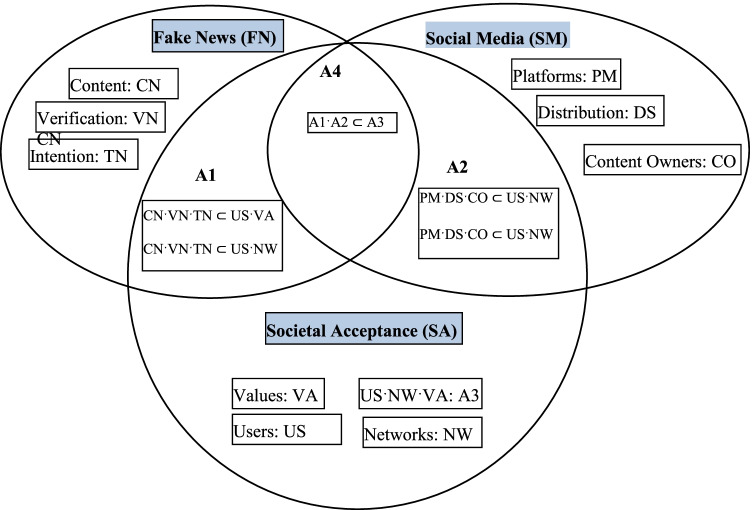
Integrated meta-framework

According to Ragin (2013); Ragin and Pennings (2005), the fuzzy set theoretical approach can be used to evaluate theories, frameworks, and models with a deductive strategy driven by a positivist paradigm. Fuzzy set analysis is an emerging technique for management and social sciences, which has become more popular as the initial problems were overcome by introducing hybrid techniques of fuzzy set logic. This study adopts the relationship and association testing suggested by Ragin (2009) to test for Boolean expressions in the fuzzy set theoretical approach of the four intersections in Fig. 2.

**Fig. 3:**
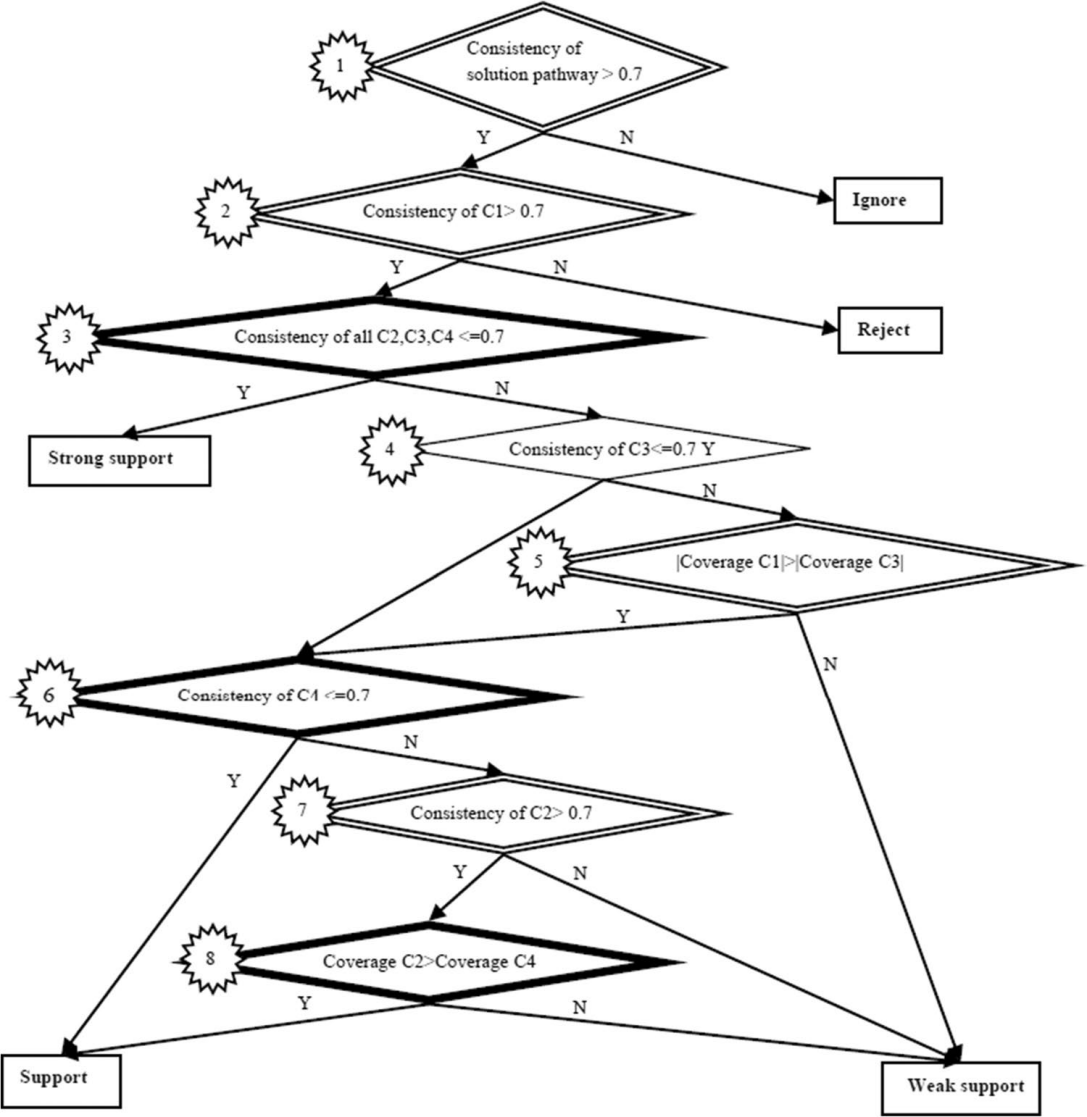
Flow chart for the consistency analysis

This study proposes an eight-step process flowchart consisting of four loop relationships (represented by the double line diamonds in Fig. 3) and three predictive relationships (represented by the single line diamonds) that shows the relationships used to discuss the outcomes of the analysis. The flowchart is described as follows:

1. A loop relationship for an expression that a solution pathway is reliable shows whether the consistency of the sufficiency analysis is greater than 0.7 of the solution pathways as defined in this paper for the consistency threshold analysis. Any relationship that falls below the set threshold is eliminated from further analysis testing, as this means that that relationship does not achieve acceptable reliability.
2. A loop relationship for an expression that a solution pathway is accepted shows whether the consistency of A1 is greater than 0.7. This statement suggests that any relationship that falls below the acceptable criteria in the solution pathway must be rejected.
3. A double line diamond relationship for a strongly supported expression shows whether the consistency of A2, A3, and A4 is less than or equal to 0.7. This statement suggests that any relationship that passes the acceptance criteria does not have significant contradictory proofs.
4. A single line diamond relationship for an expression not supported by itself (however, subsequent relationships can benefit) can be described by the consistency of A3, which is less than or equal to 0.7. Furthermore, A3 represents the type I consistency error, and it is usually below the acceptance threshold.
5. A loop relationship for an expression that a solution pathway is weakly supported shows whether the consistency of the sufficiency analysis that A1 is greater than A3 of the solution pathways, as defined for the consistency threshold analysis. Any relationship that falls below the set threshold is eliminated from further analysis, as the relationship does not achieve acceptable reliability.
6. A double line diamond relationship for a supported expression shows whether the consistency of A4 is less than or equal to 0.7. This statement suggests that any relationship that passes the acceptance criteria does not have a significant error during analysis and this supports classification.
7. A loop relationship for an expression that a solution pathway is not weakly supported shows whether the consistency of A2 is greater than 0.7. This statement suggests that any relationship that falls below the acceptable criteria in the solution pathway can be improved and there is weak support for classification.
8. A double line diamond relationship for a supported expression shows whether the consistency of A2 is greater than or equal to A4. This statement suggests that any relationship that passes the acceptance criteria and partially supports the conditions for A2 and A4 represents the type II consistency error; this is usually equal to or greater than the acceptance threshold.

## Data Analysis and Results

According to Deutsch and Malmborg (1985), complementarity and equifinality, the two underlying features in the fuzzy set theoretic approach, display patterns of attributes and different results depending on the structure of the constructs. In addition, the attributes in the constructs are concerned with the present or absent conditions and associations formed during conceptualization, rather than isolating the attributes from the constructs. Furthermore, complementarity exists if there is proof that causal factors display a match in their attributes and the analysis shows a higher level in the results, while equifinality exists if at least two unidentical pathways known as causal factors show the same results (Herrera-Restrepo et al., 2016).

In Table 3, the attributes of the constructs indicate the relationships that provide empirical evidence to reject or support the model. The results demonstrate that the relationships are mostly rejected. We find that a higher consistency level directly results in a higher reliability of the relationship. The three combinations of attributes in the sufficiency analysis show that the input efficiency either fails or passes the set consistency threshold requirement (consistency and coverage are 0.72 and 0.44, respectively).

**Table 3:** Results for A1: CN-VN-TN/USˑVA/USˑNW

	A1: FN/USˑVA			A1: FN/USˑNW
Condition	S1	S2	S3	S1
Consistency	**0.724529**	**0.713514**	**0.704821**	**0.900405**
Raw coverage	0.229618	0.209680	0.183706	0.022014
Unique coverage	0.137127	0.107350	0.069850	0.022014
Solution consistency	**0.718015**			**0.900405**
Solution coverage	0.437901			0.022014
C1: H•S⊂Y -Consistency	0.539667	0.545450	0.622072	**0.808104**
C1: H•S⊂Y -Raw coverage	0.043730	0.043524	0.036555	0.003689
C2: ~H•S⊂Y -Consistency	***0.722497***	***0.713185***	***0.703511***	***0.890097***
C2: ~H•S⊂Y -Raw coverage	0.227479	0.210136	0.183932	0.022590
C3: H•~S⊂~Y - Consistency	***0.814957***	***0.814957***	***0.814957***	0.651971
C3: H•~S⊂~Y -Raw coverage	0.112421	0.112421	0.112421	0.100733
C4: ~H•~S⊂Y -Consistency	0.463812	0.478831	0.485383	0.523584
C4: ~H•~S⊂Y -Raw coverage	0.837649	0.873858	0.891719	0.934861
Solution pathway result	Reject	Reject	Reject	Support
Combined solution pathway unique coverage of same result	0.314327			0.022014
Overall result	**Reject**			**Support**

The bold entries indicate impact of the findings and are used to further the discssion section

In Table 4, the relationships indicate support for the empirical findings. The results show that the attributes of the constructs have higher combined solution pathways than the attributes in Table 3. The type II error (or false negative) is one form of contradiction ignored in Fig. 3. These findings show the least likely attributes of the constructs, indicating the continuation of existing relationships as well as supporting the higher consistency level of the associations and stronger support for further relationships. Hence, this analysis can introduce additional causal conditions of similar attributes not yet shown in the current relationships by retracking to the relationship mapping data and finding common attributes in existing constructs. This may explain the undefined variance in the existing relationships.

**Table 4:** Results for A2: PM-DS-CO/USˑVA/USˑNW

	A2: SM/USˑVA				A2: SM/USˑNW			
Condition	S1	S2	S3	S4	S1	S2	S3	S4
Consistency	0.625760	0.693128	**0.772698**	**0.752416**	0.663176	**0.724664**	**0.794016**	**0.709135**
Raw coverage	0.479140	0.226493	0.172121	0.172026	0.098641	0.159101	0.110858	0.055455
Unique coverage	0.238754	0.069801	0.002659	0.002450	0.040192	0.074229	0.019843	0.002375
Solution consistency	0.602613				0.688200			
Solution coverage	0.554164				0.242285			
C1: H•S⊂Y -Consistency	**0.782081**	**0.873616**	**0.775306**	**0.728530**	0.674924	**0.778808**	**0.824348**	**0.711809**
C1: H•S⊂Y -Raw coverage	0.056821	0.052946	0.054630	0.057186	0.050152	0.054088	0.043457	0.056607
C2: ~H•S⊂Y -Consistency	0.625714	0.692681	***0.771952***	***0.779462***	0.678735	***0.723961***	***0.793858***	***0.763640***
C2: ~H•S⊂Y -Raw coverage	0.478587	0.226085	0.171354	0.171172	0.100992	0.158391	0.109838	0.056607
C3: H•~S⊂~Y - Consistency	0.666045	0.666045	0.666045	0.636616	0.670967	0.681394	0.681394	0.663628
C3: H•~S⊂~Y -Raw coverage	0.072447	0.072447	0.072447	0.063638	0.071768	0.075269	0.075269	0.069434
C4: ~H•~S⊂Y -Consistency	0.538359	0.532113	0.526908	0.527574	0.536492	0.536244	0.537995	0.530698
C4: ~H•~S⊂Y -Raw coverage	0.623064	0.842742	0.894709	0.896900	0.936302	0.897471	0.934667	0.967440
Solution pathways result	Ignore	Ignore	Support	Support	Ignore	Support	Support	Support
Combined solution pathway unique coverage of result			0.005109			0.096447		
Overall result	**Support**				**Support**			

The bold entries indicate impact of the findings and are used to further the discssion section

Table 5 shows the combined solution pathways for consistency and coverage, indicating support for most of the attributes of the constructs. This indicates a type I error (or false positive) in the form of contradicting the variances in the relationships, while the higher consistency level of the associations supports the higher values that delimit the relationships. Therefore, unconfirmed attributes indicate a restriction of the current relationships.

**Table 5:** Results for A3: A1-A2/USˑVA/USˑNW

	A3: A1ˑA2 /USˑVA					A3: A1ˑA2/USˑNW		
Condition	S1	S2	S3	S4	S5	S1	S2	S3
Consistency	**0.714269**	**0.745312**	**0.756022**	0.673542	**0.760762**	**0.821701**	**0.769282**	**0.849219**
Raw coverage	0.272201	0.131173	0.196403	0.265147	0.070395	0.259547	0.284802	0.266998
Unique coverage	0.137118	0.037563	0.005708	0.054258	0.002810	0.051003	0.076259	0.060114
Solution consistency	0.660851					**0.802112**		
Solution coverage	0.477160					0.395919		
C1: H•S⊂Y-Consistency	**0.901349**	**0.819554**	**0.823014**	**0.816842**	**0.760675**	**0.865469**	**0.850784**	**0.862620**
C1: H•S⊂Y -Raw coverage	0.063707	0.072059	0.067632	0.084341	0.071578	0.071564	0.087208	0.069849
C2: ~H•S⊂Y -Consistency	***0.715188***	***0.762347***	***0.755263***	0.673175	***0.816064***	***0.821572***	***0.760743***	***0.849751***
C2: ~H•S⊂Y -Raw coverage	0.272237	0.134220	0.195520	0.263983	0.071578	0.256083	0.270314	0.266000
C3: H•~S⊂~Y - Consistency	***0.910560***	***0.907633***	***0.910560***	***0.910560***	***0.905573***	0.529645	0.595851	0.520320
C3: H•~S⊂~Y -Raw coverage	0.086160	0.083161	0.086160	0.086160	0.081162	0.054214	0.054214	0.054214
C4: ~H•~S⊂Y -Consistency	0.474625	0.472827	0.471777	0.481787	0.458589	0.478524	0.473277	0.463005
C4: ~H•~S⊂Y -Raw coverage	0.876657	0.976411	0.934270	0.900039	0.989185	0.813244	0.787465	0.786341
Solution pathway result	Support	Support	Support	Ignore	Support	Support	Support	Support
Combined solution pathway unique coverage of result	0.183199					0.187376		
Overall result	**Support**					**Support**		

The bold entries indicate impact of the findings and are used to further the discssion section

In Table 6, this combined solution pathway indicates that neither the predicted relationships nor the coverage by attributes’ definitions of the constructs are strongly supported in terms of societal acceptance and the challenges posed by FN on SM on society. Therefore, alternative variances, as understood by the society, are better-supporting conditions for the relationship’s definitions in A4. Five of the six pathways are equal to or greater than the defined threshold, indicating that the relationships between the constructs can benefit from trade-offs. Furthermore, there are similar results for the unique coverage, signaling a significantly high-efficiency input directly linked to the variance from the causal conditions.

**Table 6:** Results for A4: A1-A2/A3

	A4: A1ˑA2/A3				A4: A1ˑA2/A3					
Condition	S1	S2	S3	S4	S1	S2	S3	S4	S5	S6
Consistency	0.648344	0.663247	**0.782438**	**0.772698**	**0.707672**	**0.724664**	**0.794016**	0.697460	**0.773250**	**0.778194**
Raw coverage	0.196212	0.374276	0.115329	0.172121	0.102809	0.159101	0.110858	0.250632	0.153986	0.033637
Unique coverage	0.054184	0.241412	0.037515	0.032313	0.032455	0.058696	0.016003	0.120965	0.028882	0.010464
Solution consistency	0.635798				**0.714627**					
Solution coverage	0.538797				0.454133					
C1: H•S⊂Y-Consistency	**0.791743**	**0.954857**	**0.796242**	**0.875266**	**0.748266**	**0.776939**	**0.833337**	0.672732	0.688173	**0.865103**
C1: H•S⊂Y -Raw coverage	0.054777	0.042356	0.059158	0.046974	0.054794	0.041098	0.039283	0.016219	0.018201	0.005915
C2: ~H•S⊂Y -Consistency	0.645642	0.663392	***0.774616***	***0.771952***	***0.721813***	***0.723961***	***0.793858***	0.697353	***0.772928***	***0.780676***
C2: ~H•S⊂Y -Raw coverage	0.192817	0.375529	0.111991	0.171354	0.102856	0.158391	0.109838	0.250811	0.154502	0.033991
C3: H•~S⊂~Y - Consistency	0.615825	0.600694	0.643375	0.600694	0.596100	0.600781	0.600781	0.600781	0.600781	0.600781
C3: H•~S⊂~Y -Raw coverage	0.046819	0.046819	0.046819	0.046819	0.044053	0.047553	0.047553	0.047553	0.047553	0.047553
C4: ~H•~S⊂Y -Consistency	0.544902	0.542449	0.517564	0.524309	0.525862	0.532296	0.532542	0.526383	0.539682	0.528046
C4: ~H•~S⊂Y -Raw coverage	0.897811	0.736226	0.933547	0.896900	0.934876	0.897471	0.937648	0.798192	0.905846	0.958520
Solution pathway result	Ignore	Ignore	Support	Support	Support	Support	Support	Ignore	Reject	Support
Combined solution pathway unique coverage of result			0.069828		0.117618				0.028882	
Overall result	**Support**				**Support**					

The bold entries indicate impact of the findings and are used to further the discssion section

To fully understand the A4 outcomes, it is important to discuss the outcomes from A1, A2, and A3 simultaneously. A1 and A2 are insufficient to support a high input efficiency, indicating that SM will fade-out without a correlation with FN. To have a high input efficiency, the combination of the two constructs is highly significant to the relationships. However, A3, which considers all the attributes in the societal acceptance constructs, rejects the associated attributes from A1, whereas it shows weak support for A2, which indicates that the conditions are peripheral or are unconcerned about the variance. This explains the weak support in the attributes of their relationships. The A4 outcome shows that this study considers the attributes of the relations between A1 and A2, as A3 can explain the outcomes of redefining and reducing the impact of both associations.

## Discussion

The aim of this research was to carry out an investigation on the impact of FN on the society, the use of SM as a platform for cascading of information and news. Thus, this study further explore the conceptual model of *disease triangle* (Piccialli et al., 2021) which identify FN as *infectious pathogen* in Fig. 1 (SM platforms host and spread FN), without the societal acceptance, it is difficult to cascade information and news. Furthermore, FN as defined in this study holds three main features which are significant for the perceptions of the society: the contents of the news, the intentions of the news, and the verification of the news. Hence, the use of comparative technique (fsQCA analysis) to outline the findings as shown in this study auggesting that societal acceptance is important in understanding the impact of FN. To better understand FN, SM, and societal acceptance, this study developed a meta-framework and analyzed the relationships among the attributes of the three constructs within. An online survey with 356 participants was carried out with a stratified sample size to test the meta-framework, and the data collected from the survey process were further categorized as the relationships designed in the constructs. This study considered SM platforms and the activities stimuling cascading processes of FN, changing the societal acceptance through the lens of contents management.

In previous studies, SM platforms are increasingly changing business activities and strategies used in positioning new products and brands, also leading to mis-information in the society (Modgil et al., 2021; Parra et al., 2021; Piccialli et al., 2021), also analyzed the SM platforms as the environment for business and social transactions focusing on capturing the largest audiences for information cascading, this further the spread of FN through the use of cascading tools available on SM. According to (Dwivedi et al., 2018; Kim & Dennis, 2019; Kim et al., 2019), cascading of FN through the use of SM platforms is growing faster than anticipated. The results of this study identified focused areas that can reduce the spread of FN on SM.

The results gathered during data analysis of validated questionnaire demonstrated important contributions of this study to minimizing cascading of FN in the society. Thus, the evaluation of the three perspectives; FN, SM, and societal acceptance further enhanced into relationship mapping by considering the entities from each perspectives as shown in Fig. 2. The results from Table 3, suggest that the testing of the relationship A1: FN/USˑVA of FN perspective and the entities users and values of the societal perspective is rejected while the relationship A1: FN/USˑNW of FN perspective and the entities users and networks of the societal acceptance is supported. Furthermore, the outcomes in Table 3 concur with the disease triangle theory which discussed the pathology model for disease manifestation, stating that the three triangular elements for infectious pathogen must be present for disease to grow (Humprecht, 2019b; Rubin, 2019; Sommariva et al., 2018). Hence, the relationship A1: FN/USˑVA of FN perspective and the entities users and values of the societal perspective lacks the environment (networks) for cascading of contents of FN.

Table 4 shows support for SM and societal acceptance perspectives relationship mapping, with constructs’ consistency and coverage meeting the set requirement in Fig. 3. However, condition S1 and S2 for A2: SM/USˑVA and S1 for A2: SM/USˑNW were ignored from the result, suggesting that there are other sources of information such as true news, entertainment contents which users are engaging with on SM platforms. According to Kwon et al. (2017), SM platforms provide positive opportunities such as learning new skills, engaging with experienced individuals and mentors, and finding new friendship, directly impacting positively on the society.

The increase in the level of cascading of FN can be attributed to SM companies drive to upsurge the size of big data, leading to strategic end to end nodes multiplication (Haigh et al., 2018). This study demonstrates that the enabling environment for the spreading of FN is attributed to the structure and strategies of SM companies. As shown in Table 6, when SM companies implement effective fact-checking tools on SM platforms, the traffic of FN is minimized and the impact on the society is reduced. The relevant role of SM companies is to ensure that verification and fact-checking are embedded into the process of retrieving news and information.

In summary, the findings of this study suggest that previous studies (Dwivedi et al., 2018; Kim et al., 2019; Malik et al., 2020; Modgil et al., 2021; Roozenbeek & van der Linden, 2019) demonstrated the gap for an investigation of the societal acceptance of contents available on SM. Our findings show that the societal acceptance of information and news is highly dependent on the verification and fact-checking features that are available on the SM platforms. Therefore, the research questions in this study outlined the need for fact-checking and verification of information and news most importantly FN on SM. The results of the complementarity assessments show that SM and societal acceptance did significantly influence cascading of contents towards users. Specifically, FN cascading spread faster than any other type of contents on SM as shown in Table 5. With regards to societal acceptance, users distributions of FN contents unconsciously aid cascasding with the intention of spreading awareness about the situation surrounding FN events.

### Theoretical Implications

This study builds on the theoretical knowledge in literature by making significant contribution to the understanding of the impact of FN and SM platforms on the society. According to studies (Abouzeid et al., 2021; Au et al., 2021; Dwivedi et al., 2018; Kim et al., 2019; Parra et al., 2021; Tran et al., 2021) with combined body of knowledge on misinformation, FN, SM, SM platforms, cascading of FN, and risks of misinformation, this study identifies three main themes in our contribution: FN, SM, and societal acceptance. Previous studies (Orso et al., 2020; Pennycook et al., 2020) have presented FN and SM concepts, however this study’s introduction of societal acceptance is a novel theoretical contribution. Furthermore, the lack of studies on the societal acceptance of cascading of FN have generated a theoretical gap in understanding FN, misinformation and SM. Therefore, the results in our paper filled the research gap by validating the proposed features of societal acceptance: users, networks, and values.

The findings of this study contribute to theory by using complementarity among FN, SM, and societal acceptance to explain their influence by evaluating all the attributes in the three constructs, building relationships, and presenting findings that identify the significance of each association to reduce the cascading of FN in society. Therefore, this research answers the call of studies (George et al., 2018; Miller & Tucker, 2013; Miranda et al., 2016) that have suggested further work on FN on SM. Further, this study explains the impact of FN on society by exploring the conditions in different scenarios and with different complementarity values. It also shows how SM (i.e., the environment) and users can strategically deploy all resources to tackle the cascading and spread of FN. Most importantly, fuzzy set theory provides a data analysis structure that shows complex causality, enabling this research to present empirical findings.

Theoretically speaking, the outcomes show the importance of fact-checking and managing cascading in reducing the spread of the contents of FN in the society. Also, the role of SM companies in continuance commitment to support the course of minizing the impact of FN. As of date, this is the first of study to develop a meta-framework to examine the impact of FN on the society distributed on the SM. This study argued that exploring fact-checking and managing cascading will provide a platform for SM companies to contributing in the challenging impact of FN on the society. This study finds that SM as a type of environment is equipped with the technological know-how to tackle the spread of FN. This is particularly so for large SM organizations such as Facebook whose main business is SM content. Therefore, investment in technological research and service innovation is becoming a priority. However, more investment is required for fact-checking and analyzing cascading news, meaning that SM organizations with technical research facilities are more likely to initiate rigorous fact-checking campaigns. Hence, profitability and market growth may be more important for implementing fact-checking and news-cascading technologies that benefit society.

### Practical Implications

Based on the outcomes obtained from the complementarity of the fuzzy set, it is also important for the SM platform providers to continue to invest in the fact-checking and managing contents of FN that are influencing users perceptions. In addition, it is very important to manage the direct impact of FN contents on the society by increasing the amount of fact-checking and verification tools that are available on SM. For instance, vigorous campaigns on the important role of news and information verification across all SM platforms and ensuring that there is educating information about the impact of spreading FN on SM on the society at large. Also, SM organizations should implement safe technology such as real-time deletion of contents of FN to ensure a safer communication environment for the users. Furthermore, the distinguishing real news from fake news using aided technology will boost confidence in the society. The comprehensive theoretical review and in-depth empirical analysis of the complex casualty of FN on SM on society in this study allows SM organizations to consider their organizational strategies to reduce FN cascading and implement sustainable solutions. SM organizations should prioritize the allocation of resources toward measures that tackle the challenges FN poses to society as well as the cost, societal impact, and misinformation linked to regulations to halt the spread of FN.

### Implications for Society

The in-depth empirical analysis conducted concerning the FN on SM and the societal impact, the study provides a platform to the SM users on how far the facts published on SM can be trusted and how to filter the FN from TN on SM. SM organizations such as Facebook and Twitter have invested in large to tackle the publishing of FN on social media while yet the FN has taken on SM drastically during certain urgent situations.

Following the countless challenges that arose around the world due to the FN published on SM and the societal impact, the SM organizations have taken larger steps in minimizing the FN before being published and open to the public. The flowchart for the consistency analysis can be used by SM organizations in analyzing the published news on SM to distinguish FN from TN. Thus, the negative impact caused by FN to users and their lives can be minimized. Despite the fact that steps been taken by the SM organizations, it is also users’ responsibility to filter TN from FN even if they are being posted on verified accounts, by fact-checking or using appropriate verification (Nagi, 2020).

## Conclusions

The results from this study demonstrate that it is important for SM platform providers continue in their efforts to understand the risks of cascading of FN and the influence on the society at large. Hence, the implementation of fact-checking tools is significant in reducing the spread of FN, building of trust and confident in the society. SM platform providers should ensure that there is continuous monitoring of online activities triggered by spread of FN and also ensures periodic upgrade of fact-checking technologies to tackle new tricks and strategies used in cascading FN in the society (Modgil et al., 2021; Parra et al., 2021). Furthermore, fact-checking information and public awareness on how to verify news can be added to campaigns to support the affected societies in combating the impact of FN. The findings in our study demonstrate that societal acceptance is a powerful tool that can persuade the society to focus on achieving common goal. The role of the society is to adopt the strength in societal acceptance to drive positive cultural change that welcome fact-checking and verification of any form of news.

### Limitations and Future Research Directions

This study, like other studies, has limitations that suggest future research directions. This study analyzed how three constructs, FN, SM, and societal acceptance, impact on society. Other constructs were not included in this study such as SM firms’ power, political strategies, and societal perceptions. In addition, our data collection focused on people who engage most frequently with SM; experts and SM analysts may be relevant for future research to examine. Given that previous researchers focus on cascading FN and fact-checking news content to distinguish TN from FN, the influence of fact-checking and analyzing FN cascading could be tested future research with new datasets. In this vein, this study did not consider the financial impact of FN on SM on society, which is another interesting area for future research.

This cross-sectional research aimed to provide an in-depth understanding of the relationships of the three studied topics by analyzing data from many demographics rather than from one location. Therefore, the findings of this study support generalization to many locations. However, since some studies consider the results from a single location, future research could compare the complementarity, consistency, and coverage of a single location with many locations, which would enrich the findings of this study.
